# Tsunami Squares: Leapfrog scheme implementation and benchmark study on wave–shore interaction of solitary waves

**DOI:** 10.1038/s41598-024-63010-1

**Published:** 2024-06-06

**Authors:** Yu Zhang, Kunlong Yin, Yang Tang, Lili Xiao

**Affiliations:** 1 Central-South Architectural Design Institute Co., Ltd. (CSADI), Wuhan, China; 2 Department of Engineering, China University of Geosciences (Wuhan), Wuhan, China; 3 Key Laboratory of Geotechnical Mechanics and Engineering of the Ministry of Water Resources, Changjiang River Scientific Research Institute, Wuhan, China; 4 School of Highway, Chang’an University, Xi’an, China

**Keywords:** Solitary wave, Benchmark, Wave–shore reaction, Eulerian–Lagrangian algorithm, Hydrology, Natural hazards

## Abstract

Impulse waves are generated by rapid subaerial mass movements including landslides, avalanches and glacier break-offs, which pose a potential risk to public facilities and residents along the shore of natural lakes or engineered reservoirs. Therefore, the prediction and assessment of impulse waves are of considerable importance to practical engineering. Tsunami Squares, as a meshless numerical method based on a hybrid Eulerian–Lagrangian algorithm, have focused on the simulation of landslide-generated impulse waves. An updated numerical scheme referred to as Tsunami Squares Leapfrog, was developed which contains a new smooth function able to achieve space and time convergence tests as well as the Leapfrog time integration method enabling second-order accuracy. The updated scheme shows improved performance due to a lower wave decay rate per unit propagation distance compared to the original implementation of Tsunami Squares. A systematic benchmark testing of the updated scheme was conducted by simulating the run-up, reflection and overland flow of solitary waves along a slope for various initial wave amplitudes, water depths and slope angles. For run-up, the updated scheme shows good performance when the initial relative wave amplitude is smaller than 0.4. Otherwise, the model tends to underestimate the run-up height for mild slopes, while an overestimation is observed for steeper slopes. With respect to overland flow, the prediction error of the maximum flow height can be limited to ± 50% within a 90% confidence interval. However, the prediction of the front propagation velocity can only be controlled to ± 100% within a 90% confidence interval. Furthermore, a sensitivity analysis of the dynamic friction coefficient of water was performed and a suggested range from 0.01 to 0.1 was given for reference.

## Introduction

Impulse waves generated by rapid subaerial landslides, avalanches, or glacier break-offs, may cause catastrophic destruction to the structures close to the shore and threaten human lives. Various studies have been conducted investigating different hydraulic processes related to impulse wave events: including wave generation, wave propagation, and wave-shore or wave-structure interaction, respectively^1–9^. These studies provide an understanding of wave magnitudes, characteristics and decay rates, which are important for hazard assessment and management.

The approaches which have been widely used for studying impulse waves are generally analytical methods, laboratory model tests, and numerical simulations. Laboratory model tests have been conducted to investigate the impulse wave generation mechanisms by different trigger sources, to identify the initial wave characteristics, to study propagation and shore/structure-wave interaction patterns and to establish empirical prediction equations^3,10–15^. Heller and Hager conducted a series of laboratory tests with impulse waves generated by free granular landslides in a 2D wave channel and proposed a single dimensionless parameter, the impulse product parameter, to describe key impulse wave characteristics and their magnitudes^16^. Physical experiments are essential to better understand the hydraulics of impulse waves. Experimental approaches involve high cost, are time-consuming, space-constrained as well as non-inclusive, and errors may result from the model geometry and potential scale effect caused by e.g. surface tension and viscosity^17–19^.

Computational Fluid Dynamics (CFD) solvers may allow for simulating impulse waves with the benefits of low cost and flexibility. In CFD, fluids are often regarded as a continuum. To obtain numerical solutions, two fundamental approaches can be adopted. The first one involves mesh-based numerical methods with an Eulerian definition of the flow field^20–22^. Models based on depth-integrated equations, such as 2D shallow water equations (SWE) and Boussinesq equations, have been widely used to study impulse wave propagation^23–25^. However, these methods may also be time-consuming because they require fine meshes and are based on more demanding numerical schemes. The same problem applies to 3D Navier–Stokes (NS) mesh-based solvers. Besides, most methods based on the Eulerian viewpoint inevitably have to handle advection terms, which are also computationally intensive^26^.

The second approach involves mesh-free particle methods with a Lagrangian definition of the flow field. For meshfree methods, a set of arbitrary particles is used to represent the nodes of the computational domain. This setting overcomes the problem of mesh distortion caused by large deformations in mesh-based methods. Smoothed Particle Hydrodynamics (SPH) represents one of these approaches and has also been applied for simulating impulse waves^27–30^. Wang et al. used SPH to simulate the impulse wave induced by landslides and obtained good agreements with observed data^29^. Nevertheless, as the time complexity of the indispensable neighbouring particle searching procedure is the square of the number of particles, the method requires a large number of particles to exhibit good wave behaviour, leading to high computational costs.

Hybrid Lagrangian–Eulerian numerical methods received attention for simulating impulse waves due to their capability to strengthen the advantages of both the aforementioned methods and avoid their disadvantages^31–33^. Tsunami Squares (TS), which is also a hybrid Lagrangian–Eulerian method, is an extension of the Tsunami Balls method introduced by Ward et al.^34^. With the characteristics of both Lagrangian and Eulerian viewpoints, TS allows for modelling hydrodynamic problems using limited computation resources^35–37^. This method has been previously applied to back analyse the entire impulse wave process chain, including landslide dynamics, wave generation, propagation and run-up, of several historical impulse waves. Xiao et al. initially introduced this method and modelled a historical impulse wave event generated by the Gongjiafang landslide in the Three Gorges area, China, in 2008^35^. The landslide’s failure pattern, wave generation, propagation and run-up were all well reproduced using a laptop computer. Wang et al. simulated the 2007 Chehalis Lake landslide and impulse waves with TS, and the results of slide mass and run-up height were in good agreement with the finding in the post-event field survey conducted by Robert et al^38,39^. With respect to the laboratory scale, Wang et al. conducted a benchmark study on the landslide-water interactions by comparing the wave characteristic calculated by TS with 3D experiments^40^. Moreover, the study included a sensitivity analysis with various ratios of wave generation parameters. They concluded that the mechanical processes of landslide-water interaction have been well represented in TS.

However, these researchers paid more attention to landslide dynamics and landslide interaction with the water body, rather than wave propagation, which is also very important for hazard assessment for shore areas. In addition, limited parameter conditions have been simulated in their studies, e.g. fixed slide impact velocity. Consequently, it is difficult to assess the simulation results’ accuracy when using TS to predict impulse waves under different parameter conditions. It is therefore essential to carry out systematic validation studies to ascertain TS’s applicability and predictive accuracy involving every stage of an impulse wave event.

Solitary waves represent waves with the largest energy content, which could steadily propagate in constant water depth, and have similar hydrodynamic characteristics compared with impulse waves, at least for the leading wave within the wave train^14,41, 42^. A large number of theoretical and experimental studies on solitary waves have been carried out so far. As a result, solitary waves are often used as the benchmark of numerical simulation methods^43–45^.

Besides, solitary waves are among the types of impulse waves obtained by Heller and Hager by analysing the wave characteristic parameters of impulse waves under different experimental conditions^18^. In the field, large mass wasting events such as rapid landslides may generate solitary-like waves^46^. From their 3D experiments, Mohammed and Fritz found that the propagation velocity of the leading wave crest corresponds closely to the theoretical approximation of the solitary wave speed^47^. In addition, Evers and Boes compiled numerous experimental data and empirical equations about solitary and impulse wave run-up heights, concluding that solitary waves could be considered a suitable proxy for assessing the run-up height of the leading wave of an impulse wave train^14^. Therefore, considering the extensive research base and the similarity with impulse waves in terms of wave characteristics, the solitary waves are often utilised as a reference to study the hydraulic properties of impulse waves^48–50^.

**Figure 1:**
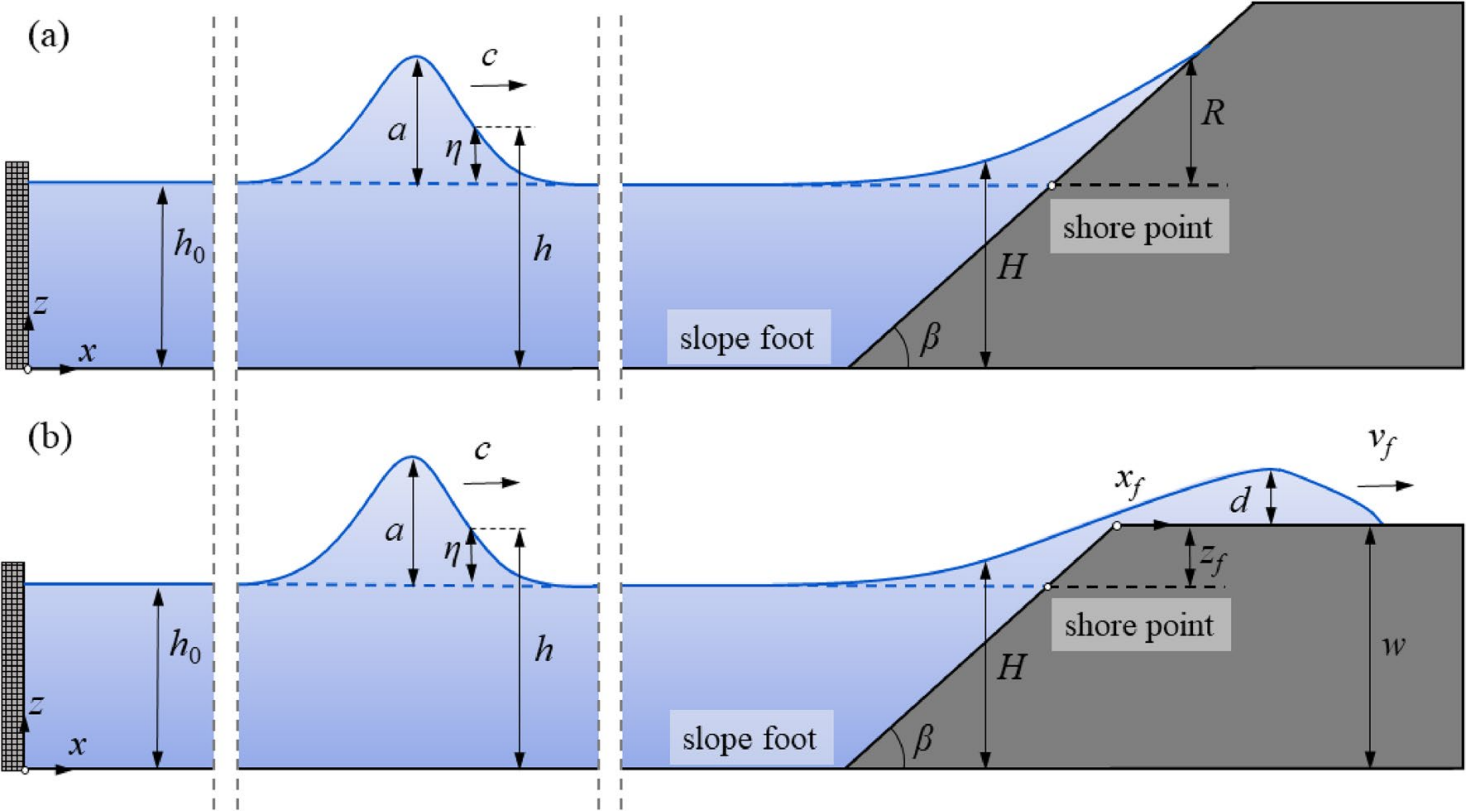
Definition scheme for solitary wave run-up (**a**) and overland flow (**b**) with governing parameters and target quantities.

This study benchmarks Tsunami Squares against laboratory experiments on run-up at a slope and induced overland flow, as well as previously published empirical formulations. The numerical wave channel and a definition sketch of solitary wave run-up and overland flow are shown in Fig. 1 with *x* as the horizontal coordinates, water surface displacement *η*, solitary wave amplitude* a* and solitary wave celerity *c*, as well as the shore slope *β*, shore height *w*, distance along horizontal shore *x*_*f*_, maximum overland flow depth *d* and overland flow front propagated velocity *v*_*f*_. The influence of *a*_0_, *h* and *β*on the predicted *R*, *d* and *v*_*f*_ are evaluated and analysed. Furthermore, a sensitivity analysis of the dynamic friction coefficient *μ*_*d*_ representing the friction induced by bottom roughness and viscosity is conducted.

## Numerical method

Tsunami Squares (TS) is a numerical method originally developed to simulate the behaviour of landslides, debris flow, water waves, and any other flow or flow-like processes. TS was proposed by Xiao et al., as an update of a similar method known as Tsunami Balls^34,35^. After its initial implementation written in Fortran, the algorithm was rewritten in C +  + by Wilson et al^51^. In TS, a continuum body is meshed into several small squares which carry the physical quantities: flow depth, density, velocity, acceleration and momentum. A single square is considered a Lagrangian element for calculating the particle’s acceleration and velocity at the current time and predicting the new position in the next time step. Then, this square becomes a *ghost* square carrying volume (height) and momentum^35^. The ghost square, considered an Eulerian element, disappears after its volume and momentum are distributed to the overlapped mesh squares. The simulation process consists of the following stages:

### Initialization of squares

**Figure 2:**
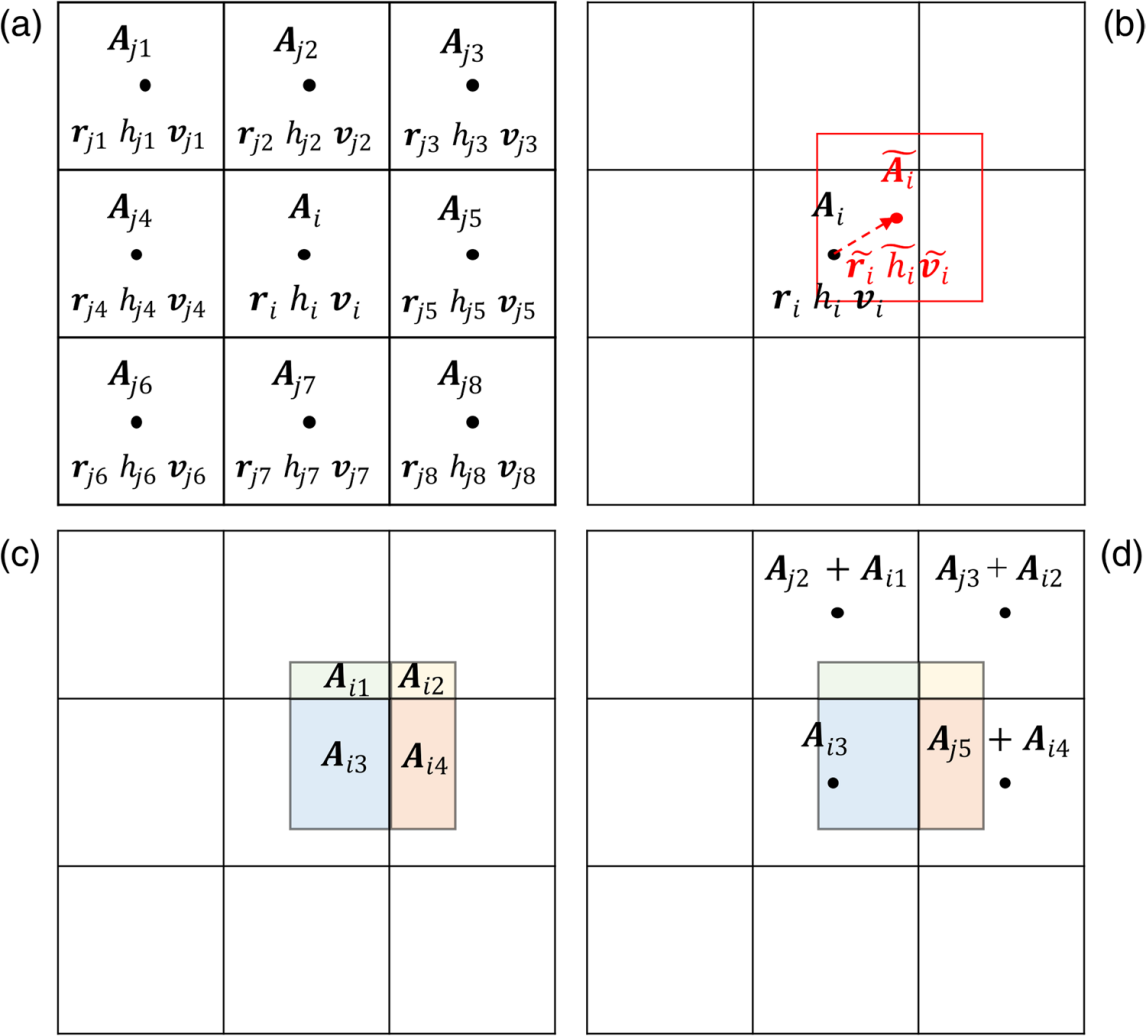
Tsunami squares wave propagation concept.

The computational domain is discretised into a collection of squares, as shown in Fig. 2a, each with its initial physical properties ***A*** (position ***r***, height *h*, velocity ***v***). The bold symbols indicate a two-dimensional vector.

### Translate the squares for the first time

The first loop begins from here. Each square’s acceleration is calculated based on the surrounding squares and rheology by summing accelerations from different sources. For water squares, gravitational and frictional acceleration are considered. The gravitational acceleration *g* is caused by the water surface gradient that includes the effect of the bed slope:

1 $${\varvec{a}}_{i\left( t \right)g} = - g\nabla H\left( t \right)$$

With *H*(*t*) is the water surface elevation including the bathymetry at time *t*, *i* represents the ID number of central squares and *j*1–*j*8 indicates the surrounding eight squares.

The frictional acceleration is calculated as

2 $${\varvec{a}}_{i\left( t \right)f} = - \mu_{d} \frac{{\left| {{\varvec{v}}_{{{\varvec{i}}\left( {\varvec{t}} \right)}} } \right|{\varvec{v}}_{{{\varvec{i}}\left( {\varvec{t}} \right)}} }}{{h_{i\left( t \right)} }}$$

With the dimensionless dynamic friction parameter *μ*_*d*_ representing all velocity-depended resistance, and the velocity of square *i* at time *t ****v***_*i*(*t*)_. Note that *μ*_*d*_ can be regarded as a tuning parameter, depending on the bed roughness and fluid viscosity, as it has no specific physical meaning^37^. Summing these two terms, the total acceleration ***a***_*i*(*t*)_ of each square is obtained and the first loop is completed.

### Translate the squares for the second time

Then the second loop starts. Since the acceleration of each square is known, the displacement of squares during a single time step can be calculated by integrating over d*t*. Combined with the initial location ***r***_*i*(*t*)_, the new location $\tilde{\user2{r}}_{l}$ at time *t* + d*t* is obtained, as shown in Fig. 2b. Note that the time step should be chosen small enough to satisfy the Courant–Friedrichs–Lewy (CFL) criterion for depth-average methods, defined as

3 $${\text{d}}t < CFL_{min} \left( {\frac{{{\text{d}}x}}{{{\varvec{v}}_{i\left( t \right)} + \sqrt {gh_{i\left( t \right)} } }}} \right)$$

With the Courant number *CFL* and d*x* representing the square size^20^.

As can be seen in Fig. 2c, square *i* is then moved to the new location $\tilde{\user2{r}}_{i}$ and becomes a ghost square. The height and momentum carried by the ghost square are distributed and merged to the four overlapping squares *j* (Fig. 2d) based on the following equations:

4 $$\delta V_{ji} = \left( {H_{i} {\text{d}}x^{2} } \right)\left( {1 - \frac{{\left| {\tilde{x}_{i} - x_{j} } \right|}}{{{\text{d}}x}}} \right)\left( {1 - \frac{{\left| {\tilde{y}_{i} - y_{j} } \right|}}{{{\text{d}}x}}} \right){ }H_{j} \left( {t + {\text{d}}t} \right) = \frac{{\mathop \sum \nolimits_{i = 1}^{N} \delta V_{ji} }}{{{\text{d}}x^{2} }}$$

5 $$M_{ji} = \left( {\rho_{w} H_{i} {\text{d}}x^{2} \tilde{\user2{v}}_{i} } \right)\left( {1 - \frac{{\left| {\tilde{x}_{i} - x_{j} } \right|}}{{{\text{d}}x}}} \right)\left( {1 - \frac{{\left| {\tilde{y}_{i} - y_{j} } \right|}}{{{\text{d}}x}}} \right)\;{\varvec{v}}_{j} \left( {t + {\text{d}}t} \right) = \frac{{\mathop \sum \nolimits_{i = 1}^{N} \delta M_{ji} }}{{\rho_{w} {\text{d}}x^{2} H_{j} \left( {t + dt} \right)}}.$$

After the distribution, the ghost square *i* disappears. The height and momentum of all squares have been updated and the second loop ends.

### Smoothing process

A smoothing process is used for every moving square to avoid non-physical artefacts of the discrete simulation. In order to obtain a stable simulation, it is necessary to execute smoothing processes once or even several times at each time step, but the smooth times, which can be considered another tuning parameter, should be used as minimally as possible. A modified smooth process is proposed based on the former studies, which is described in Sect. “Smooth function”^37,51,52^. Then the flow computation can proceed to step (2) to continue the next time step or end the programme if the pre-set time limit is exceeded.

In TS, the material domain is discretised by Lagrangian squares carrying physical information which can be moved to a new position the integration over time is accomplished based on the Lagrangian perspective. However, the exchange of volume and momentum between the square and neighbouring squares is performed based on a mesh-based background, which provides an Eulerian description of the domain. In comparison to a general numerical method based on particles, the square particles used in TS whose neighbouring particles can be obtained directly from coordinates, eliminate the need for neighbourhood search algorithms. Relative to grid-based simulation algorithms, TS replaces the convection term with the redistribution of the physical quantities of the ‘ghost’ square. Based on the above two characteristics, TS is capable of simulating flow and flow-like processes efficiently.

## Updated numerical scheme

### Leapfrog scheme

The Leapfrog time-stepping scheme that emerged from the early years of numerical weather prediction is a popular method for solving shallow water equation^53^. One of its advantages is that it preserves exactly the amplitude of a pure oscillation^54^. A new algorithm proposed by Altaie and Dreyfuss using the Leapfrog scheme and Centre Finite Differences was used to solve 2D shallow water equations and the test results showed high performance^55^. Puthenveettil and Mandli compared the Euler and the Leapfrog scheme on Nonlinear Shallow Water Equations and found that the Leapfrog time-stepping scheme produced a more accurate solution than the Euler method^56^.

In TS, the position and velocity of the ghost square are calculated based on an Euler scheme (Eqs. (6), (7)).

6 $$\tilde{\user2{r}}_{i} \left( t \right) = {\varvec{r}}_{i} \left( {t - {\text{d}}t} \right) + {\varvec{v}}_{i} \left( {t - {\text{d}}t} \right)dt + 0.5{\varvec{a}}_{i} \left( {t - {\text{d}}t} \right){\text{d}}t^{2}$$

7 $$\tilde{\user2{v}}_{i} \left( t \right) = {\varvec{v}}_{i} \left( {t - {\text{d}}t} \right) + {\varvec{a}}_{i} \left( {t - {\text{d}}t} \right){\text{d}}t$$

$\tilde{\user2{r}}_{i}$ and $\tilde{\user2{v}}_{i}$ represent the new position and new velocity of the ghost square, respectively. Considering the advance of the Leapfrog scheme, we applied it to calculate time integration and named the implementation Tsunami Square-Leapfrog (TSLF) to achieve second-order accuracy. Equation (7) is replaced by the following equation:

8 $$\tilde{\user2{v}}_{i} \left( t \right) = {\varvec{v}}_{i} \left( {t - {\text{d}}t} \right) + \frac{{{\varvec{a}}_{i} \left( t \right) + {\varvec{a}}_{i} \left( {t - {\text{d}}t} \right)}}{2}{\text{d}}t$$

In the meantime, the $\tilde{\user2{v}}_{i}$ in Eq. (6) was replaced by the $\tilde{\user2{v}}_{i}$ calculated by Eq. (8) in TSLF.

### Smooth function

The smoothing process involves the redistribution (smoothing) of the height and momentum of one square in comparison with its adjacent squares (after Fig. 1d). For each neighbouring square, the water height *f*_sm_|*h*_i_-*h*_j_| is taken from the higher square to the lower square. The weighting factor is generally considered to be related to the thickness of the water squares, which is smoothed more where the water is deeper and less where the water is shallower. Besides, It is necessary to define a maximum value of *f*_*sm*_ to avoid too much volume or momentums being distributed during the smoothing process. For example, Eq. (9) was used by Wilson et al. to decide the weighting factor *f*_*sm*_ in the smoothing process^51^.

9 $$f_{sm} = 0.15\;{\text{min}}\left( {0.02 + 0.125\left( {\frac{{h_{i} }}{6000}} \right),0.5} \right)$$

Since the smoothing process has to be executed at least once per time step, a variation in time steps inevitably leads to a variation in execution times of smoothing processes in the same time period, which can affect the smoothness. Moreover, as the smoothing procedure acts on a constant number of squares instead of a constant area, the square size also directly affects the smoothing range. It is infeasible to employ Eq. (9) for convergence tests, as the same smoothness cannot be guaranteed in a smooth process with a different temporal or spatial resolution. In addition, Eq. (9) is not in a dimensionless form. In summary of the above, a new weighting equation is proposed for the smoothing process:

10 $$f_{sm} = {\text{min}}\left( {0.45{\text{d}}t\sqrt {\frac{g}{{{\text{d}}x}}} e^{{ - \frac{{{\text{d}}x}}{{h_{i} }}}} ,0.225} \right)$$

The new weighting equation eliminates the aforementioned shortcomings and is applicable to different scales. Both the time step and square size were taken into consideration; therefore, the new weighting equation is able to keep a constant smoothness during convergence tests.

The smoothing function prevents the non-physical behaviour of the waves, but it reduces the wave’s potential energy at each time step, which may cause excessive wave attenuation, especially for long propagation distances^52^. Therefore, on the one hand, we need to apply as small smoothness as possible to maintain numerical stability. On the other hand, any potential energy lost to the system is compensated by increasing the velocities of each square to ensure that the system’s total energy remains constant. After computing the smoothed quantities, the original values are replaced.

## Results

### Convergence tests

A solitary wave propagating on a flat bottom wave channel with a width of 2 m and a length of 15 m was simulated by TSLF to investigate the convergence and inspect the performance of the new smoothing Eq. (10). According to Boussinesq^57^ the solitary wave profile for a finite wave length is

11 $$\eta \left( {x,t} \right) = a{\text{sech}}^{2} \left( {\sqrt {\frac{3a}{{4h_{0}^{3} }}} \left( {x - x_{0} - ct} \right)} \right),$$

With the amplitude *a* and the initial position of the wave crest *x*_0_^57^. The depth-averaged horizontal velocity is $\tilde{v}_{x} = \frac{c\eta }{{h_{0} + \eta }}$ while the vertical velocity is zero. For practical purposes, an effective solitary wave length $L_{95\% } = \frac{{4.24h_{0} }}{{\sqrt {a/h_{0} } }}$ containing 95% of the wave volume, is used in the present study^42^.

**Figure 3:**
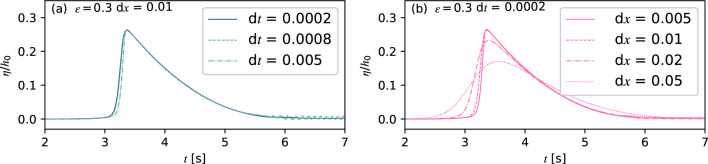
Wave profile convergence tests for solitary waves at *x* = 7.68 m. (**a**) The comparison of wave profile under the same *ε* = 0.3 and d*x* = 0.01 with different d*t*. (**b**) The comparison of wave profile under the same *ε* = 0.3 and d*t* = 0.0002 with different d*x*.

The still water depth *h*_0_ was set to 0.2 m, the initial relative wave amplitude *ε* = *a*/*h*_0_ = 0.3, and the initial position of wave crest amplitude is at *x* = 3.5 m. Figure 3 shows the comparison of wave profiles at *x* = 7.68 m from models running with different time steps and square sizes. Results from d*t* = 0.0002 s and 0.0008 s are similar for d*x* = 0.01. While small oscillations appear behind the wave crest for d*t* = 0.005 s with *CFL* = 0.9, which was considered the maximum value to obtain stable results. Therefore, as long as d*t* is less than 0.005 s, the magnitude of the time step will have a negligible effect on the results. d*t* = 0.0002 s was chosen to obtain more detailed time-dependent results. Afterwards, four square sizes, d*x* = 0.05 m, 0.02 m, 0.01 m and 0.005 m, were adopted for this case. The present models with d*x* = 0.02 m and 0.05 m underpredicted the wave amplitude by 11.54 and 34.61%, respectively, compared with d*x* = 0.01 m. The deviation between the results for d*x* = 0.005 m and 0.01 m is less than 0.1%, indicating a convergence with grid refinement for d*t* = 0.0002 s. Hence, the final time step and square size were set to be d*t* = 0.0002 s and d*x* = 0.01 m for all the following simulations.

In the meantime, it was easy to find that excessive smoothness could lead to a reduction in accuracy, so the smallest smoothness that leads to stable results should be used in the simulation.

### Comparison of TS and TSLF

A few numerical simulations for solitary waves were conducted to compare the performance of the original model TS and the new model TSLF. Firstly, solitary waves propagating in a two-dimensional frictionless channel with a constant depth were used as a benchmark for TS and TSLF.

The length of the numerical channel is 15 m and the width 2 m. The domain was discretized with a constant spacing d*x* = 0.01 m, and the time step d*t* was set to 0.0002 s (*CFL* = 0.036), based on the convergence tests. The still water depth *h*_0_ was set to 0.2 m. The initial position of the wave crest was at *x* = 2 m + *L*_95%_. The simulation duration was set to 9 s which was sufficient for the wave to propagate for at least 25*h*_0_. The simulations included seven different initial relative wave crest amplitudes ranging from 0.05 to 0.7.

**Figure 4:**
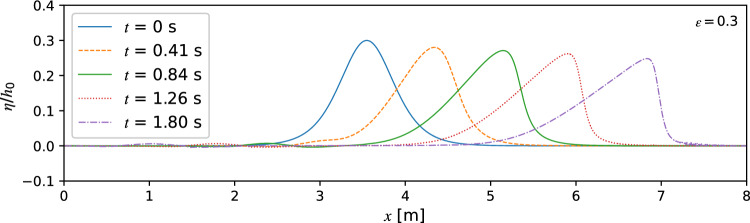
Snapshots for the wave profiles of a solitary wave propagating in a constant depth wave channel using TSLF.

Snapshots of the free water wave surface with *ε* = 0.3 at *t* = 0 s, 0.41 s, 0.84 s, 1.26 s, and 1.80 s are shown in Fig. 4. Only the results of TSLF are shown for brevity. The wave crest amplitude increases by 13.1% in the beginning and reaches a peak value of 0.41 s. From 0 s to 0.84 s, the initial solitary wave profile separated into a large wave crest and a much smaller wave trough. After the separation, the wave profile becomes asymmetric with a steeper wavefront. In addition, the wave amplitude is decreasing with increasing propagation distance.

**Figure 5:**
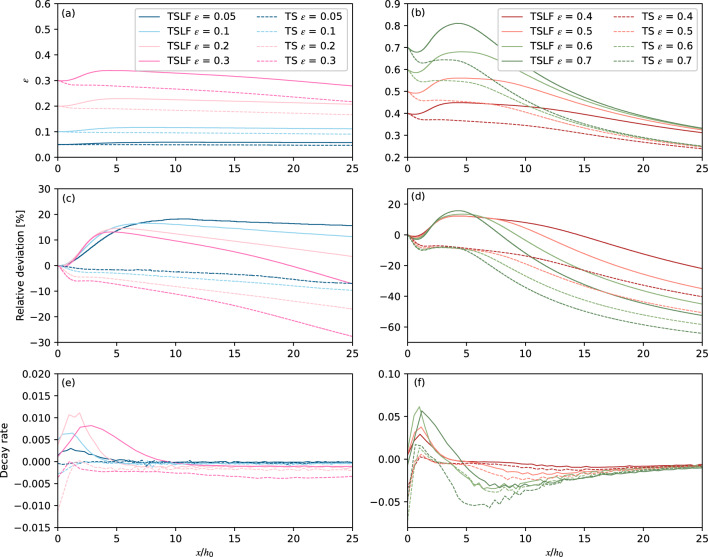
Relative wave amplitude, relative deviation and decay rate over initial wave amplitude changes with relative propagated distance. (**a**) *ε* changes with the relative propagation distance when *ε* ≤ 0.3. (**b**) *ε* changes with the relative propagation distance when *ε* > 0.3. (**c**) The relative deviation rates of *ε* from the initial relative wave amplitude change with the relative propagation distance when *ε* ≤ 0.3. (**d**) The relative deviation rates of *ε* from the initial relative wave amplitude change with the relative propagation distance when *ε* > 0.3. (**e**) The decay rates of *ε* from the initial relative wave amplitude change with the relative propagation distance when *ε* ≤ 0.3. (**f**) The decay rates of *ε* from the initial relative wave amplitude change with the relative propagation distance when *ε* > 0.3.

The relative wave crest amplitudes *ε* (a, b), their deviation rate from the initial value *ε* (c, d) 100% × (*ε*-*ε*_0_)/*ε*_0_, and the decay rate (e, f), which was calculated by $\frac{\varepsilon \left(t+\text{d}t\right)-\varepsilon (t)}{\left[x\left(t+\text{d}t\right)-x(t)\right]/{h}_{0}}$, for all simulations are shown in Fig. 5. For both methods, the wave crest amplitude increased to the peak first and then decreased monotonically during propagation. The larger the wave, the stronger the wave amplitude decay. Note that the smoothing process was executed once for the tests in Fig. 5a, while it had to be executed twice for the cases in Fig. 5b to get stable results. Even though, a small oscillation still existed for *ε* = 0.7.

The notable differences in wave amplitudes between TS and TSLF are already present during the initial increasing process. The wave amplitude increased by around 10–20% for TSLF, while it decreased for TS in the near field (*x*/*h*_0_ < 2.15). This results in the wave amplitude of TSLF being larger than TS on one hand. On the other hand, the decay rates of TSLF, as shown in Fig. 5e, f, were lower than for TS for the same propagation distance. For example, the decay rate of *ε* = 0.3 between *x*/*h*_0_ = 15 to 20 is 0.35% for TSLF, while it is 0.55% for TS. Thus, TSLF slightly overestimates the wave amplitude in the near field, but could provide a lower decay rate in the far field compared to TS. Furthermore, the increase in the wave amplitude in the near-field is considered to be of minor importance, as it is the result of the input wave equation not being matched to the kernel of TS and TSLF. It is reasonable to believe that this phenomenon does not occur when the wave is generated by mass movement.

For the tests with initial *ε* > 0.4, their relative wave amplitudes are gradually approaching 0.4 with increasing propagation distance. Given that, TSLF and TS are suitable to simulate the wave propagation process when the relative wave amplitude is smaller than 0.4. Applied to large waves (*ε* > 0.4), both TS and TSLF are likely to underestimate wave amplitudes with increasing propagation distance.

In order to comprehensively evaluate the performance of TS and TSLF, we compared them with experiments for solitary wave run-up along a slope and induced overland flow. As shown in Fig. 4, the waveform becomes asymmetrical during propagation, therefore to use a waveform closer to the solitary wave, the initial wave crest amplitude position was set to a distance of *L*_95%_ (*L*_95%_/*h*_0_ = 7.74) away from the slope foot.

**Figure 6:**
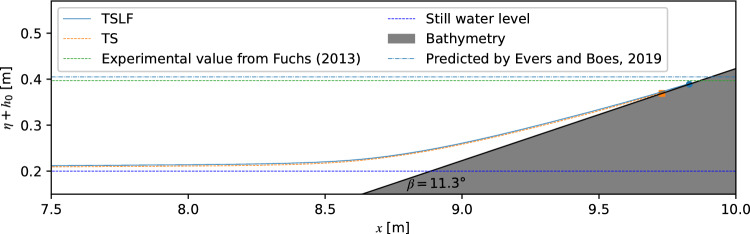
Comparison of water surface profile at the moment of reaching the highest point and wave run-up height between experimental data and different numerical methods.

Figure 6 shows the wave profiles when reaching the highest elevation for *h*_0_ = 0.2 m, *ε* = 0.3 and *β* = 11.3 ^∘^. Numerical results were compared with the observed run-up height value *R* = 197 mm in the experiments of Fuchs for the same condition^42^. Given the accuracy of the experiments, 1 mm was used to distinguish the inundated squares from dry squares. Based on this criterion, the maximum run-up height was marked in Fig. 6. The water surface profiles of TS and TSLF at this moment were close. For the run-up height, however, a discrepancy of 20 mm was observed between TS and TSLF. The numerical model TSLF slightly underestimated the measured wave run-up height by − 4.11%, while TS underestimated the measurements by − 14.26%. Therefore, the results from both methods were in acceptable agreement with the experiment, while TSLF appears to have a slightly better performance than TS. However, as shown in Fig. 5a for solitary wave propagation over a horizontal bottom, TS and TSLF yield different initial *ε* = 0.276 and 0.334, respectively, at the slope foot.

**Figure 7:**
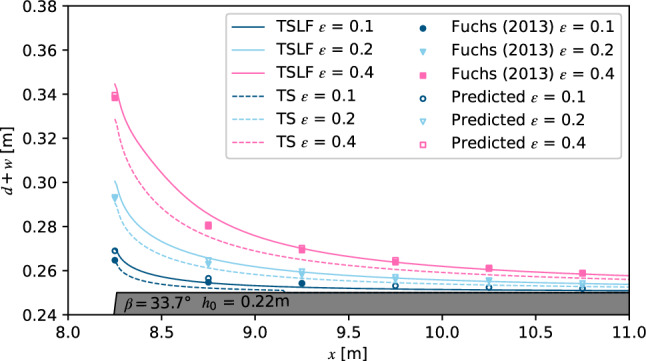
Comparison of maximum overland flow depth along freeboard between experimental data and numerical simulations.

We then reproduced the experiments by Fuchs on solitary wave induced overland flow using both TS and TSLF^42^. Six wave gauges spaced by 0.5 m were installed above the free broad to catch the maximum overland flow depth *d* in the physical experiments (Fig. 1). The maximum *d* along the horizontal shore during the whole process was recorded in the numerical simulation and the empirical equations proposed by Fuchs were also used for comparison^42^. The result for *h*_0_ = 0.22 m, *β* = 33.7 ^∘^ and *ε* = 0.1, 0.2 and 0.4 is shown in Fig. 7. It can be found that *d* from the updated method TSLF were greater and closer to the observed value than for the method TS. In fact, the differences between TS and TSLF already exist when the waves arrive at *x*_*f*_ = 0 m (the point where the run-up process transitions to overland flow). The *d* at *x* = 8.25 m calculated by TSLF exceeds TS by 30.8%, 20.79% and 17.46% for *ε* = 0.1, 0.2 and 0.4, respectively, indicating that the difference of *d* between TSLF and TS has been made during the wave propagation and run-up process before the overland flow process. Most of the observed maximum flow depth *d* was located between TS and TSLF. Given particular importance for engineering applications, TSLF, which could give a safer result, is considered a suitable tool for wave run-up and overland flow process simulation.

### Solitary wave run-up

**Figure 8:**
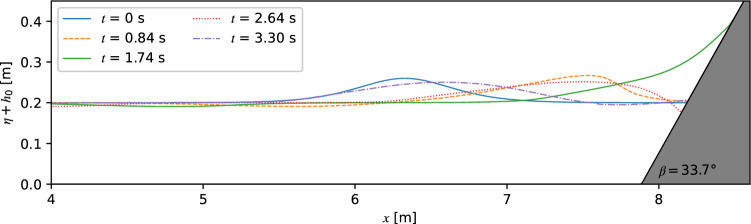
Solitary wave run-upfor *β* = 33.7°, *h*0 = 0.2 m and *ε* = 0.3.

A wide range of slope angles *β* = 5°, 11.3°, 21.8°, 33.7°, 45° and 70° and initial wave amplitudes *ε* = 0.05, 0.1, 0.2, 0.3, 0.4, 0.5, 0.6 and 0.7 were considered in the numerical simulation of solitary wave run-up. Figure 8 shows snapshots of a solitary wave running up a slope of *β* = 33.7° for *ε* = 0.3, *h*_0_ = 0.2 m at different times. The initial wave crest amplitude position was set to a distance of ${L}_{95\%}$ from slope foot, as shown in Fig. 8. The solitary wave started to run-up the slope after 0.84 s, reached the highest elevation at 1.74 s, and ran down and created a steep wave trough at 2.64 s. At 3.30 s, the reflected wave has developed and is propagating away from the slope.

#### Wave run-up height

**Figure 9:**
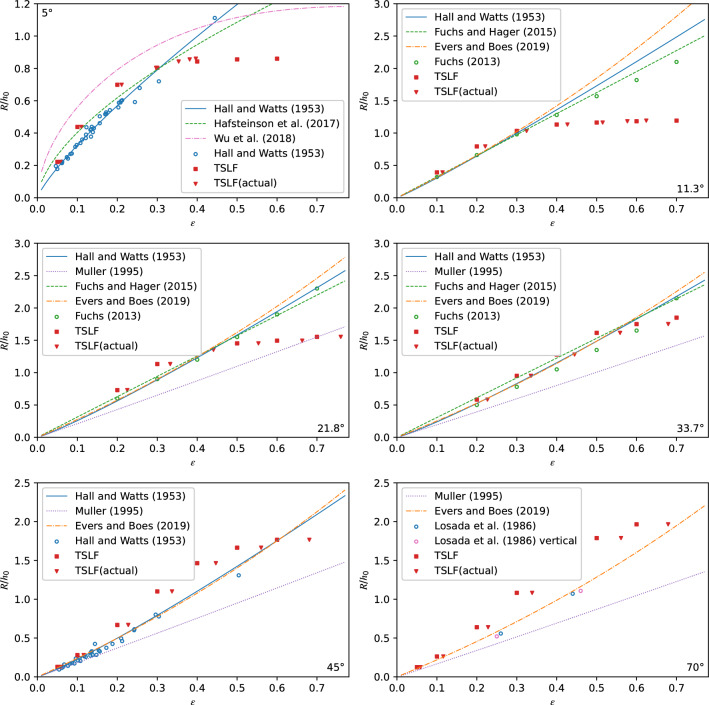
Comparison of the relative run-up height *R* calculated by TSLF, predicted by empirical equations, and measured by Hall and Watts^59^ and Fuchs^42^ under the scenario of (**a**) *β* = 5°, (**b**) *β* = 11.3°, (**c**) *β* = 21.8°, (**d**) *β* = 33.7°, (**e**) *β* = 45°and (**f**) *β* = 70°.

To assess the accuracy and performance of the numerical method TSLF, the simulation results were compared with empirical equations and experimental data. 19 experiments from Fuchs and Hager considering three slope angles (11.3 ^∘^, 21.8 ^∘^, 33.7 ^∘^), 69 experiments from Hall and Watts including two slope angles (5 ^∘^ and 45 ^∘^) and 4 experiments from Losada et al. for 70° and 90° were plotted in Fig. 9^58–60^. Furthermore, the empirical equations in Table 1 used for predicting wave run-up heights were included according to their applicability range.

**Table 1:** Empirical equations included in the analysis.

References	Empirical equations	Applicability range
Hall and Watts^59^	$\frac{R}{{h_{0} }} = 11\left( {\beta \frac{\pi }{180^\circ }} \right)^{0.67} \varepsilon^{{1.9\left( {\beta \frac{\pi }{180^\circ }} \right)^{0.35} }}$	5–12°
	$\frac{R}{{h_{0} }} = 3.05\left( {\beta \frac{\pi }{180^\circ }} \right)^{ - 0.13} \varepsilon^{{1.15\left( {\beta \frac{\pi }{180^\circ }} \right)^{0.02} }}$	12–45°
Müller^61^	$\frac{R}{{h_{0} }} = 1.25\left( {\frac{90^\circ }{\beta }} \right)^{0.2} \varepsilon^{1.25} \left( {\frac{\varepsilon h}{L}} \right)^{ - 0.15}$ with $L_{Sol} = \frac{{2\pi h_{0} }}{{\sqrt {0.75\varepsilon } }}$	18.4–90°
Fuchs and Hager^58^	$\frac{R}{{h_{0} }} = 3\left( {{\text{tan}}\beta } \right)^{ - 0.05} \varepsilon$	11.3–33.7°
Hafsteinsson et al.^64^	$\frac{R}{{h_{0} }} = 19{\text{tan}}\beta \varepsilon^{{({\text{tan}}\beta )^{0.2} }}$	1–6°
Wu et al.^65^	$\frac{R}{{h_{0} }} = \frac{{4.50\left( {\xi_{s} } \right)^{1.52} }}{{0.04 + \left( {\xi_{s} } \right)^{1.19} }}$ $\xi_{s} = {\text{tan}}\beta \varepsilon^{ - 0.9}$	*ξ*_*s*_ < 1.20
Evers and Boes^14^	$\frac{R}{{h_{0} }} = 2\varepsilon e^{0.4\varepsilon } \left( {\frac{90^\circ }{\beta }} \right)^{0.2}$	10–90°

The relative run-up height versus initial wave amplitude results calculated by TSLF are shown as red squares in Fig. 9. As noted previously, the wave amplitude of a solitary wave calculated by TSLF changes during propagation. Consequently, the wave amplitude propagating for the same distance from the initial position to the shore point in a constant depth wave channel is presented as the red triangle (named actual wave amplitude) for reference.

For the mild shore, i.e. *β* ≤ 21.8°, the relative wave run-up heights are in good agreement with experimental data and empirical equations for the initial wave amplitudes for *ε* < 0.4. For *ε* > 0.4, however, the numerical method increasingly underestimates the run-up height. It is because the initial wave is too large (Fig. 5) for TSLF to maintain the wave amplitude during the long propagation distance caused by a mild shore. Especially for 5° and 11.3°, the actual wave amplitude (the red triangles) was concentrated around* ε* = 0.4, although the initial wave amplitude was larger. Note that for 5°, the solitary wave may break if ε > 0.129 and become a plunging or surging breaker during the run-up process according to the breaking criterion proposed by Grilli et al^62^. Pudjaprasety developed a method to couple the staggered scheme shallow water equation and SPH and used the coupled model to simulate the run-up of a solitary wave with a relative initial wave amplitude of 0.3 along a slope with an angle of 2.86°^63^. the predicted results overestimated the run-up height by 5.83% compared to the experiments conducted by Synolakis^41^. This performance was similar to the TSLF’s performance when simulating a solitary wave running up along a slope with an angle of 5°.

The empirical equation for breaking solitary waves proposed by Hafsteinsson et al. and Wu et al. is also shown in Fig. 9. Wroniszewski et al. presented a benchmark of four available solvers for the Navier–Stokes equation: Gerris, OpenFOAM, Thétis and Truchas. The numerical run-up height was compared with an empirical formula to evaluate the performance of the four solvers^44,64,65^. They also found that the average deviation grows with the height of the wave. For the slope angle of 10°, the deviations of run-up height computed by the best solver were around 10% for *ε* = 0.1, whereas they were around 30% for *ε* = 0.3. Relatively speaking TSLF has better performance than these four solvers. Besides, Their simulations reveal that the computed runup heights are much closer to the results predicted by the empirical formula than in the case of the 10° beach, which was consistent with the performance of TSLF. As seen in Fig. 9, TSLF could give a reasonable estimation of the run-up height when *ε* < 0.4, which contains the range where the waves may break, even though wave breaking is not considered in TSLF.

For the steep shore, i.e. *β* ≥ 33.7°, the numerical results overestimated *R* compared to the experimental data and empirical equations. Regarding *R* at a vertical shore, it follows a very closed pattern with 70° according to Losada et al. (see circles in Fig. 9)^60^. In addition, the run-up height decreases by 5% from 70° to 90° according to Evers and Boes^14^. Thus, the relative run-up height is only slightly affected by the shore angle changing from 70° to 90°. Considering TSLF cannot simulate abruptly changing bathymetry such as a 90° slope; we recommended changing vertical shores to 70° shores if necessary and adding a safety margin of X%.

In summary, it was concluded that the numerical method TSLF could give a reasonable or safe prediction for all shores when the initial wave amplitude *ε* is smaller than 0.4. Meanwhile, TSLF may underestimate the run-up height at mild slopes for *ε* > 0.4.

#### Wave reflection

**Figure 10:**
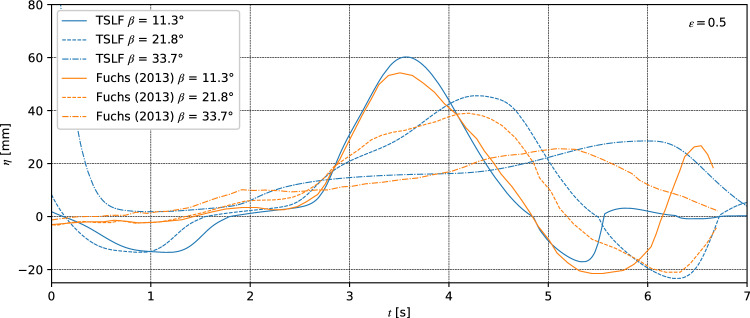
Run-up wave reflection for various slope angles.

To study wave reflection and dissipation of the numerical method, the tests of Fuchs were simulated using TSLF^42^. Numerical gauges were set at the exact location with the experiments to record the free water surface time series. Among them, the gauge USD 0 located at *x* = 5.8 m (in front of the shore) was used to observe the reflected wave. Details of the experimental setup are provided in Fuchs^42^. Numerical results of free surface elevations versus time at the gauge UDS 0 for *ε* = 0.5 and *β* = 11.7°, 22.3° and 33.7° were compared with observed results (Fig. 10). The wave shape of numerical simulations and the experiments matched well as indicated by Fig. 10. Concerning the experiment for the steep shore (*β* = 33.7°), the reflected wave was composed of a pronounced leading wave, followed by a parent secondary wave. For the numerical result, the wave amplitude of the leading wave was 6.8 mm over the experimental value, while the wave peak of the secondary wave was significantly smaller. In contrast with the steep shore, reflection is more gradual for gentle shores, causing a long-flatted reflected wave. The reflected wave amplitude of *β* = 11.3° and *β* = 22.8° calculated by TSLF was 11.2 mm and 3.14 mm larger than the experimental values, respectively.

**Figure 11:**
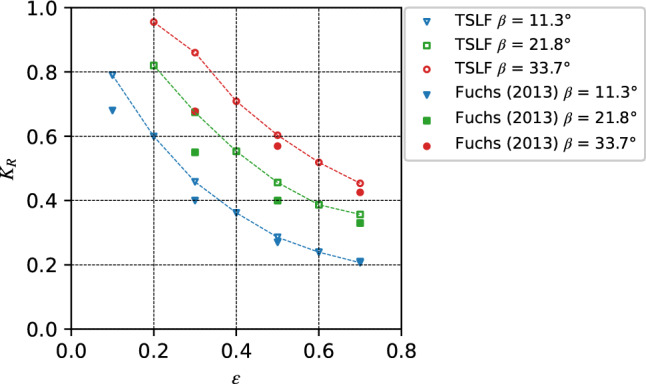
Comparison of the wave reflection coefficient of different slope angles between TSLF and experiments of Fuchs^42^.

As the wave height is of particular importance for engineering applications, *K*_*R*_, as the ratio between the reflection and the initial wave crest amplitude, is considered a suitable description of the wave shore interaction processes^2,42^. Figure 11 shows the numerical versus experimental *K*_*R*_ for three different angles and different initial wave amplitudes. The numerical results generally overestimated the *K*_*R*_ with those obtained in the physical tests. Relatively speaking, steep shores and gentle shores with larger initial waves were in better agreement with experimental data.

### Overland flow

**Figure 12:**
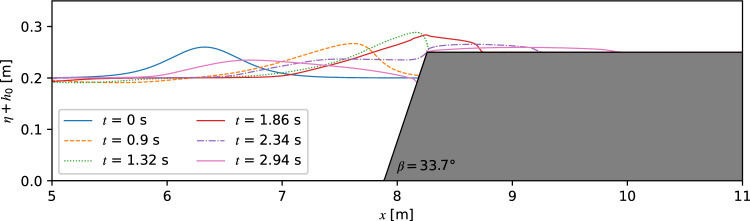
Solitary wave overland flow for *β* = 33.7°, *h*_0_ = 0.2, *ε* = 0.3.

A series of solitary waves induced overland flow in a physical water wave channel for different still water depths from 0.16 to 0.22 m, three shore angles of 11.3°, 22.8°and 33.7° and initial relative wave amplitudes from 0.1 to 0.7 were carried out by Fuchs and Hager^58^. To verify the numerical method on overland flow, the physical experiments were reproduced using TSLF. Since the shore height was fixed, the variable still water depth caused the changing freeboard and relative shore height. Snapshots of the water surface profile for *β* = 33.7 ^∘^, *h*_0_ = 0.2 m and *ε* = 0.3 at six different times are plotted in Fig. 12. The same as in the wave run-up simulation, the initial position of the solitary waves are set to a distance of *L*_95%_ from the slope foot. The solitary wave approaches the shore at 0.9 s, reaches the freeboard after 1.32 s, and has transitioned into overland flow at 1.86 s. The flow has been separated into two parts: overland flow and wave run-down after 2.34 s. The reflected wave fully developed and propagated backwards, while the overland flow front reached midway of the freeboard and continually moved forwards at 2.94 s.

**Figure 13:**
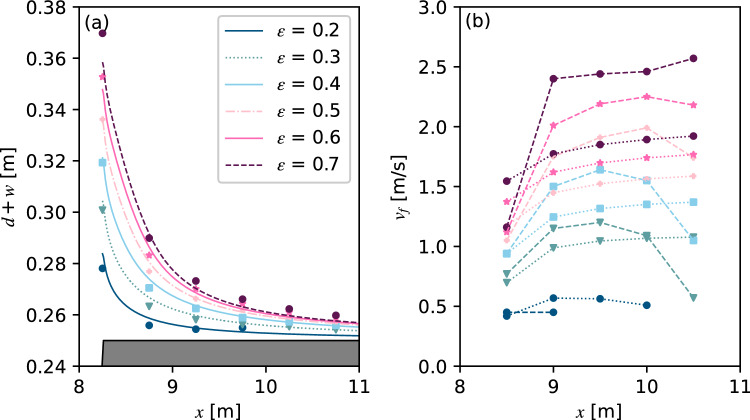
Comparison of the maximum elevation of the water surface of the overland flow and front velocity calculated by TSLF and observed by Fuchs^42^. (**a**) The maximum elevation of the water surface changes with the propagation distance under different *ε*. (**b**) The front velocity of the overland flow changes with the propagation distance under different *ε*. The colour represents the initial wave amplitude; the circle means the observed results; the square represents the numerical results.

The inundated depth and flow velocity are the main parameters for a detailed hazard assessment, including inundation areas and the determination of forces on structures. Thus, the following analysis focused on the maximum flow depth *d* along the horizontal overland flow portion and flow front propagation velocity *v*_*f*_. Note that *v*_*f*_ here is calculated by the difference in arrival time (the time when the flow depth exceeds 1 mm) of two neighbouring gauges. The numerical and experimental results are summarised in Fig. 13 for *h*_0_ = 0.2 m and *β* = 33.7°. In general, the results showed that the numerical method TSLF gives a satisfactory prediction of the maximum flow depth. Specifically, the flow depth was slightly overestimated when the relative initial wave amplitude was smaller than 0.6. As the flow propagation distance increases, the flow depth of large initial waves gradually approaches the value when *ε* = 0.4, consistent with the solitary wave pattern for run-up at a mild shore. Concerning flow front velocity, the discrepancies between numerical (squares) and observed (circles) values are not negligible, especially when *ε* > 0.4.

**Figure 14:**
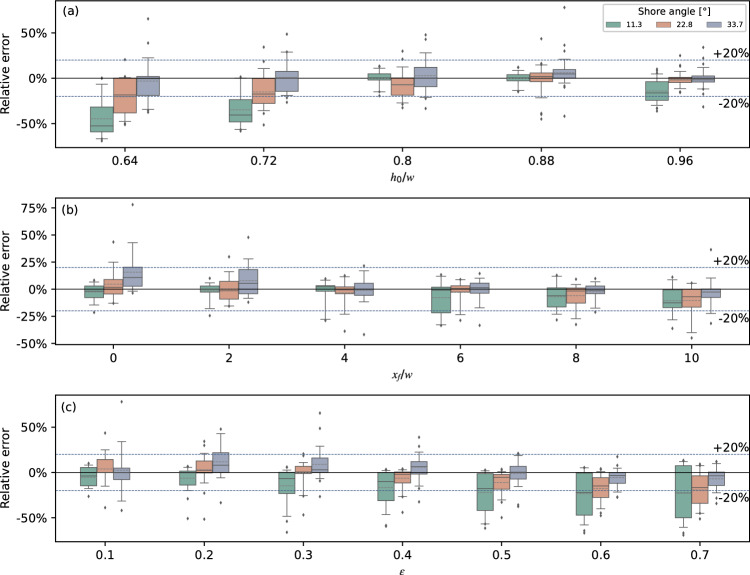
Box plots with whiskers at 5th and 95th percentiles for numerical over experimental overland flow depth versus still water depth (**a**), propagation distance (**b**) and relative initial wave amplitude (**c**) for three different slopes angles.

Based on the substantial overland flow data covering a wide range of conditions containing different still water depths, slope angles and initial wave amplitudes in Fuchs, a correlation analysis of the discrepancy between numerical and experimental results was conducted^42^. Figure 14 shows the deviation of the numerical overland flow depth over the experimental value for different angles; The solid line represents the median value, and the dashed blue line is marked ± 20% for reference.

The deviation versus still water depth is shown in the upper panel. It can be seen that the predicted deviation was basically less than 20% when the still water depth is deeper than 0.2 m. Taking the case for *h*_0_ = 0.24 m and *β* = 33.7° as an example, 90% of the data’s relative deviation was between − 11.75% and 11.83%, with a median deviation of − 1.71%. While the numerical method TSLF obviously underestimated the overland flow height when *h*_0_/*w* < 0.2 m, the deviation rate increases with decreasing *h*_0_. Fuchs and Hager^19^ found that for *h*_0_ = 0.1 m and *ε* < 0.4, the relative maximum flow depth *d*/*h*_0_ increased compared with *h*_0_ = 0.2 m during propagation along the horizontal shore due to surface tension. Hence the data for still water depths 0.16 m and 0.18 m may be influenced by scale effects, thus were removed from the following boxplots Fig. 14b and c.

The middle panel shows the deviation changes with propagation distance along the horizontal shore. It can be seen that the agreement was best in the middle of the horizontal shore. For *x*_*f*_/*w* = 0 and 2, the numerical method tends to overestimate the flow depth whilst the flow depth tends to be underestimated for *x*_*f*_/*w* > 6; 75% of the numerical results were smaller than the experimental results. The deviation for *x*_*f*_/*w* = 0 and 2 may raised by the transition from solitary wave to the overland flow. It could be found that during the transition phase, the contours of the velocity of the water were not absolutely vertical from Fuchs’s experimental study in 2013. Therefore TSLF, a numerical method based on a depth averaging algorithm, may produce inaccuracy when modelling the transition phase. As for *x*_*f*_/*w* > 6, the flow depth heights decreased with the increasing propagation distance. The surface tension has a prominent impact on the flow with a small depth, which caused the underestimation of the simulation results.

The lower panel shows that TSLF has a better performance when 0.3 < *ε* < 0.5. The deviation for a small initial wave varies significantly. In fact, the overland flow depth for small *ε* is quite small. For example, the observed *d*/*h*_0_ are 0.66, 0.21 and 0.19 for the experiment with *h*_0_/*w* = 0.88, *ε* = 0.1, and *β* = 33.7 ^∘^ at *d* = 0 , 2.00, and 4.00 respectively. And the overland flow has not reached the last UDS (*x*_*f*_/*w* = 10). Fuchs found that when the flow front came to rest along the overland flow portion and did not reach the last UDS, the flow front disintegrates into individual ‘flow fingers’ due to surface tension effects caused by small flow depths^42^. Hence the unstable deviation for small initial wave amplitude may be caused by surface tension.

The pattern is unclear regarding the shore angle, even the deviation indeed varies with it. Overall, the deviation between the numerical method and experiments was associated with propagation distance and *ε*. TSLF can accurately predict the maximum height of overland flow when propagating a short distance along the horizontal shore and with a small initial water wave. Otherwise, the overland flow depth may be underestimated, but the relative error will not exceed ± 20%.

**Figure 15:**
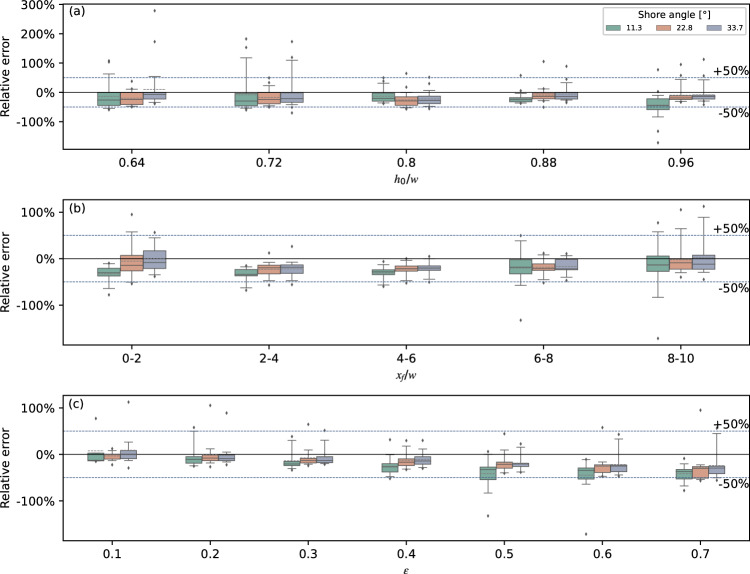
Box plots with whiskers at 5th and 95th percentiles for numerical over experimental overland flow front propagated velocity versus still water depth (**a**), propagation distance (**b**) and relative initial wave amplitude (**c**) for three different slope angles.

Figure 15 represents the boxplot for the deviation rate of numerical over experimental overland flow front velocity for different shore angles, similar to Fig. 14. For *h*_0_ < 0.2 m, the prediction of *v*_*f*_ is better than *d*. Still, the data was dropped for the middle and lower panels when *h*_0_ < 0.2 m due to scale effects. Generally speaking, the flow front velocity calculated by TSLF was smaller than the observed values. Moreover, the deviation increases with *ε*. While, the agreement improves for *x*_*f*_*/w* > 6, which is due to the flow front velocity reduction caused by turbulence in the experiments, which can be seen in Fig. 13.

To sum up, 90% of the deviation errors for maximum flow depths did not exceed ± 50%, and the ones for the flow front velocities did not exceed ± 100%. When *ε* in front of the shore was less than 0.4, TSLF could assess the inundation area and intensity reliably.

## Discussion

Solitary wave run-up along slopes covered by different materials has been studied previously by Teng et al^66^. They investigated the effect of the beach roughness on wave run-up through experiments. Three beach angles (10°, 15° and 20°) and two artificial rough beaches with a manning coefficient of 0.018 and 0.024 were used in their study. Their results showed that the effect of slope roughness on solitary wave run-up depends on both roughness and *β*. The viscosity and roughness effects are negligible for steep slopes, for which the reduction rate was 1.9–7.4% for 20°. In contrast, the run-up height could be reduced by about 33.4–35.7% for a mild slope (10°). Gedik et al. conducted an experimental study about the solitary wave run-up along a 1:5 slope (11.3°) with impermeable and permeable beaches (sandy and armoured beaches)^67^. They found that armour units cause a 50% decrease in run-up height. Arana performed solitary waves run-up a 10° slope with four types of surface, including smooth impermeable, rough impermeable and rough permeable^68^. It was found that the relative run-up height for rough impermeable beaches could be 15–27% smaller compared with the smooth impermeable beach.

**Figure 16:**
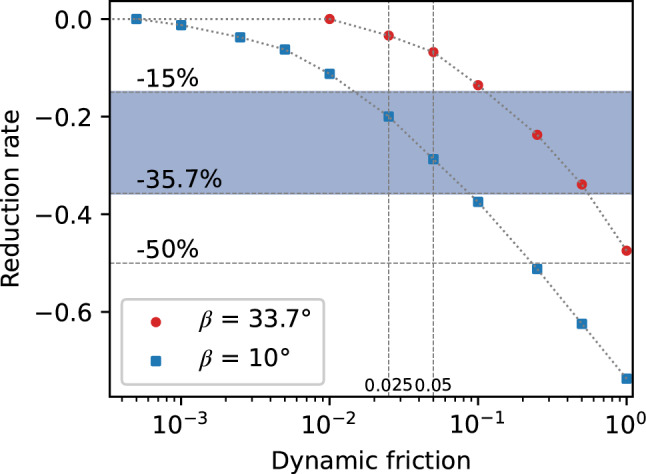
Reduction of maximum run-up height due to dynamic friction.

A sensitivity analysis is carried out to gain more insight into the dynamic friction parameter *μ*_*d*_. Therefore, additional simulations of solitary wave run-up were conducted with *h*_0_ = 0.2 m, *β* = 10° and 33.7°. These tests are shown in Fig. 16 with a reduction rate of run-up height with dynamic friction relative to no dynamic friction. For the steep shore, when *μ*_*d*_ = 0.01, the results were the same as for no friction. As the dynamic friction increased, the relative run-up height gradually decreased, and the rate of descent progressively grew. When *μ*_*d*_ = 1.0, the relative run-up height is reduced to nearly half of the original level. For the mild angle, when *μ*_*d*_ = 0.0005, the relative run-up height was the same as no friction. Apparently, the dynamic friction has a stronger influence on the run-up height for the mild shore than the steep shore, which is consistent with Teng et al^66^.

Taking the above experimental results into account, a value range for the dynamic friction coefficient from 0.01 to 0.1 was recommended, ensuring a certain influence on mild slopes meanwhile a negligible influence on steep slopes.

## Conclusions

This study proposed an updated scheme called Tsunami Squares-Leapfrog (TSLF) and developed a systematic benchmark study regarding the solitary wave propagation in a wave flume with a uniform water depth, run-up along a slope and induced overland flow under various initial conditions by comparisons with experiments and empirical equations. Moreover, a sensitivity analysis of the dynamic friction coefficient was first conducted. The main findings are as follows:

1. A new smoothing function was proposed and the time-integrated method Leapfrog was applied in Tsunami Squares. The updated numerical scheme TSLF was confirmed to be able to achieve a convergence analysis and reduce the decay rate of the wave amplitudes with propagation distance, which resulted in a smaller error between the simulated and experimental values compared with Tsunami Squares.
2. TSLF can handle the simulation of the solitary wave propagation process when the relative wave amplitude is smaller than 0.4. Otherwise, it is likely to reduce an underestimate of the wave amplitude.
3. When the shore angle *β* ≤ 21.8°, the maximum run-up height can be precisely predicted by TSLF while the initial relative wave is smaller than 0.4, however, it is underestimated while the initial relative wave exceeds 0.4. When *β* ≥ 33.7°, TSLF tends to give a safe prediction with respect to the maximum run-up height. Besides, TSLF can give a satisfactory estimation of the wave reflection coefficient.
4. TSLF can reliably assess the maximum flow depth and front propagation velocity of the overland flow reduced by solitary waves. Specifically speaking, 90% of the deviation between the simulated and experimental results for maximum flow depth can be confined to ± 50%, and the deviation for the front propagation velocity can be confined to ± 100%.
5. A range of dynamic friction coefficients from 0.01 to 0.1 was suggested as a reference for the simulations considering bottom roughness.

## Data Availability

The datasets generated during the current study are available from the corresponding author on reasonable request.
